# Global Alcohol Harm Network: Struggling or Emerging? A Response to Shiffman

**DOI:** 10.15171/ijhpm.2017.31

**Published:** 2017-03-01

**Authors:** Sally Casswell

**Affiliations:** Social and Health Outcomes Research and Evaluation (SHORE), SHORE & Whariki Research Centre, College of Health, Massey University, Auckland, New Zealand.

## Dear Editor,

Shiffman’s^1^ editorial on global health networks is a useful addition to a relatively neglected area. The nature of global health networks is an important contributor to influence in the global health arena. However, in this editorial the necessarily brief overview of each of eight topics gives a limited picture and does not do justice to the many contextual differences which influence status in the global governance arena and, perhaps more importantly, the status and nature of the health networks themselves.

It is certainly possible to draw a strong contrast between the Global Alcohol Policy Alliance (GAPA) and the Framework Convention Alliance, despite the comparable levels of harm experienced globally, and many reasons underpin this difference. One of the more influential has been the role of the industry. Since the widespread commercialisation of the production of both commodities the transnational alcohol corporations, learning from the adverse experience of tobacco, have done an excellent job of framing the problem definition to suit their ends (the contested framing mentioned by Shiffman^1^ is more between industry and public health networks rather than within or between them). They have succeeded in influencing sympathetic governments and this despite longstanding evidence of the industry’s subversion of alcohol research^2,3^ and effective alcohol policy (eg, Bakke and Endal^4^). Another important influence is a relative lack of informed advocates at national level and funding opportunities at both national and global level. This reflects the diffuse nature of alcohol related harm which makes a causal contribution to 60 diseases, and with much harm outside of the traditional health arena (for example impacts on productivity, violent crime and educational achievement); this has reduced the likelihood of support from any particular quarter, unlike the involvement of cancer and heart health charities in tobacco control. This relative lack of funding is one of the main factors inhibiting the development of the alcohol network.

Despite this, considerable progress has been made in recent years and it is not now accurate to characterise the GAPA network as consisting ‘predominantly of researchers from North American and European institutes’ (although these may have been the majority of interviewees in the 2011–2012 research,^5^ on which Shiffman’s^1^ editorial is based). GAPA has active regional networks, particularly active in Africa which is one of the key foci for the transnational alcohol corporations at the present time. The 2015 biennial conference of the GAPA attracted participants from 60 countries, many from LMICs and funded by development agencies. In 2016 GAPA worked with several member states to ensure the challenges raised by the proliferation of alcohol marketing in the digital world was discussed in a Side Event at the World Health Assembly, and the need for a legally binding international health response was raised.

There is much to do and Shiffman’s analysis^1^ is useful in reinforcing the need to extend our focus on coalition building beyond the health sector and the need for transparency in our processes. The need to move beyond an ‘emerging network’ as GAPA, until recently, described itself on its website (http://globalgapa.org/) is urgent, given the ambitions of the transnational alcohol producers in the emerging economies of the world. Their expansion is assisted by the proliferation of economic agreements weakening national governments’ role in protecting their populations and the expansion of alcohol marketing in the global digital world. This is why GAPA has determined to advocate for a stronger response in the form of an international binding treaty to control alcohol supply, marketing and the role of the industry in alcohol policy development.

Signed by GAPA Board Members:

Professor Sally Casswell, GAPA Chairperson, Massey University, New Zealand

Professor Isidore Obot, GAPA Vice Chair, School of Public Health and Policy, Morgan State University, Baltimore MD, USA and Adjunct Professor of Psychology University of Uyo, Nigeria

Professor David Jernigan, GAPA Chair Scientific Committee, Johns Hopkins Bloomberg School of Public Health, USA

Øystein Bakke, GAPA Secretary, FORUT—Campaign for Development and Solidarity, Norway

Dr. Pham Thi Hoang Anh, Country Director, HealthBridge, Vietnam

Dr. Kumnuan Ungchusak, Board Member, Thai Health Promotion Foundation, Thailand

Savera Kalideen, Advocacy Manager, Soul City Institute, South Africa

Ms. Paula Johns, REDEH – Human Development Network; founder and director of the ACT Tobacco Control Alliance, Brazil

Mr. Derek Rutherford, past Chair of the Global Alcohol Policy Alliance, UK

Professor Thomas F Babor, University of Connecticut School of Medicine, USA

Mr. Sven-Olov Carlsson, past President, IOGT International

Professor Sungsoo Chun, Sahmyook University, South Korea

Mr. George Hacker, past Director, Alcohol Policies at Centre for Science in the Public Interest, USA

Professor Ronaldo Laranjeira, Federal University of São Paulo, UNIFESP, Brazil, and Alcohol and Drug Research Unit of UNIAD

Professor Charles Parry, Alcohol & Drug Abuse Research Unit, South African Medical Research Council, South Africa

## Ethical issues

Not applicable.

## Competing interests

Author declares that she has no competing interests.

## Author’s contribution

SC is the single author of the paper, but all board members read and approved the letter.
